# Percutaneous nephrolithotomy guided by 5G-powered robot-assisted teleultrasound diagnosis system: first clinical experience with a novel tele-assistance approach (IDEAL stage 1)

**DOI:** 10.1186/s12894-024-01400-3

**Published:** 2024-01-18

**Authors:** Jie Yang, Xiang Zhou, Xuan Zhou, Jin-yong Tian, Muhetaer Wubuli, Xin-hua Ye, Jie Li, Ning-hong Song

**Affiliations:** 1 Department of Urology, First Affiliated Hospital of Nanjing Medical University, Nanjing, Jiangsu 210029 China; 2 Department of Urology, People’s Hospital of Xinjiang Kizilsu Kirgiz Autonomous Prefecture (Xinjiang Kezhou People’s Hospital), Artux, Xinjiang 845350 China; 3 Department of Ultrasound Diagnosis, First Affiliated Hospital of Nanjing Medical University, Nanjing, Jiangsu 210029 China

**Keywords:** Intraoperative tele-assistance, 5G technology, Robot-assisted, Teleultrasound diagnosis system, Complex kidney stones, Percutaneous nephrolithotomy

## Abstract

**Background:**

To demonstrate the technical feasibility of percutaneous nephrolithotomy (PCNL) guided by 5G-powered robot-assisted teleultrasound diagnosis system (RTDS) in a complex kidney-stone (CKS) cohort and present our preliminary outcomes. PCNL is highly skill-required, which hinders it popularization in primary medical units of remote regions. We designed an innovative tele-assistance approach to make PCNL easy to be operated by inexperienced surgeons.

**Methods:**

This was a prospective proof-of-concept study (IDEAL phase 1) on intraoperative tele-assistance provided by online urological experts via a 5G-powered RTDS. Total 15 CKS patients accepted this technology. Online experts manipulated a simulated probe to assist unskilled local operators by driving a patient-side robot-probe to guide and monitor the steps of access establishment and finding residual stones.

**Results:**

Median total delay was 177ms despite one-way network-connecting distance > 5,800 km. No perceptible delay of audio-visual communication, driving robot-arm or dynamic ultrasound images was fed back. Successful tele-assistance was obtained in all cases. The first-puncture access-success rate was 78.6% with a one-session SF rate of 71.3% and without complications of grade III-V.

**Conclusions:**

The current technology based on 5G-powered RTDS can provide high-quality intraoperative tele-assistance, which has preliminarily shown satisfactory outcomes and reliable safety. It will break down a personal competence-based barrier to endow PCNL with more popular utilization.

**Trial registration:**

The study was approved by ethics committee of the Xinjiang Kezhou People’s Hospital and ethics committee of the First Affiliated Hospital of Nanjing Medical University and was registered on http://www.chictr.org.cn (ChiCTR2200065849, 16/11/2022).

**Supplementary Information:**

The online version contains supplementary material available at 10.1186/s12894-024-01400-3.

## Background

Urinary stone disease is worldwide prevalent disease. Epidemiological investigations reveal its incidence range from 7 to 13% in North America, 5–9% in Europe, and 1–5% in Asia [1–3]. Due to an arid climate and high-mineral surface water as well as a high-fat diet, the prevalence of kidney stones can reach 7.47% in Xinjiang Kizilsu Kirgiz Autonomous Prefecture (Kezhou), the westernmost part of China [4, 5]. Here, patients with complex kidney stones (CKS, defined as Guy’s Stone Scores [GSS] grade III-IV) are particularly common [4, 6] and need to be treated routinely with percutaneous nephrolithotomy (PCNL).

PCNL has been a mature procedure to treat nephrolithiasis for decades and the core step is to establish proper percutaneous accesses to renal collecting system under an ultrasound or fluoroscopy guidance [7, 8]. However, this step is hard to master because of variations of stone distribution and patient’s anatomy, and requires a long learning-curve for beginners [7]. At present, only some large-center urological experts can complete it well in China. Lack of skilled urologists in local primary medical units has greatly hindered PCNL popularization, which forms a prominent contradiction with the high incidence of nephrolithiasis in the region [4, 5].

In recent years, a rapid progress in network and robot technologies has boosted the realization of telesurgery [9, 10], especially after the advent of the 5th generation mobile communication technology (5G) [11]. In 2021, we firstly applied the platform of 5G-powered robot-assisted teleultrasound diagnostic system (RTDS) to provide a high-quality intraoperative assistance for establishing percutaneous renal accesses [12]. To date, the first 15 CKS cases have been successfully treated under this technology. The aims of this study are to demonstrate technical feasibility and report preliminary outcomes. We expect the surgical innovation to play an enlightening role in overcoming the underlying inequity of medical resource distribution among regions with different development levels.

## Methods

### Study design

This was a prospective proof-of-concept study according to IDEAL criteria [13]. Tele-assistance and PCNL procedures were performed by the First Affiliated Hospital of Nanjing Medical University and Xinjiang Kezhou People’s Hospital, respectively. The study was approved by ethics committee of the Xinjiang Kezhou People’s Hospital and ethics committee of the First Affiliated Hospital of Nanjing Medical University and was registered on http://www.chictr.org.cn (ChiCTR2200065849, 16/11/2022). After adequately informed that this was the first clinical application of the technology, 14 patients and a 14-year-old patient’s guardian gave their informed consents, and all details, images, or videos relating to an individual person in this study were published after obtaining informed consent from participants. The primary endpoint of the study was to evaluate the success rate of access establishment under a tele-assistance in CKS patients; the secondary endpoints included assessments on complications and stone-free (SF) rates.

### Inclusion criteria

**Fig. 1:**
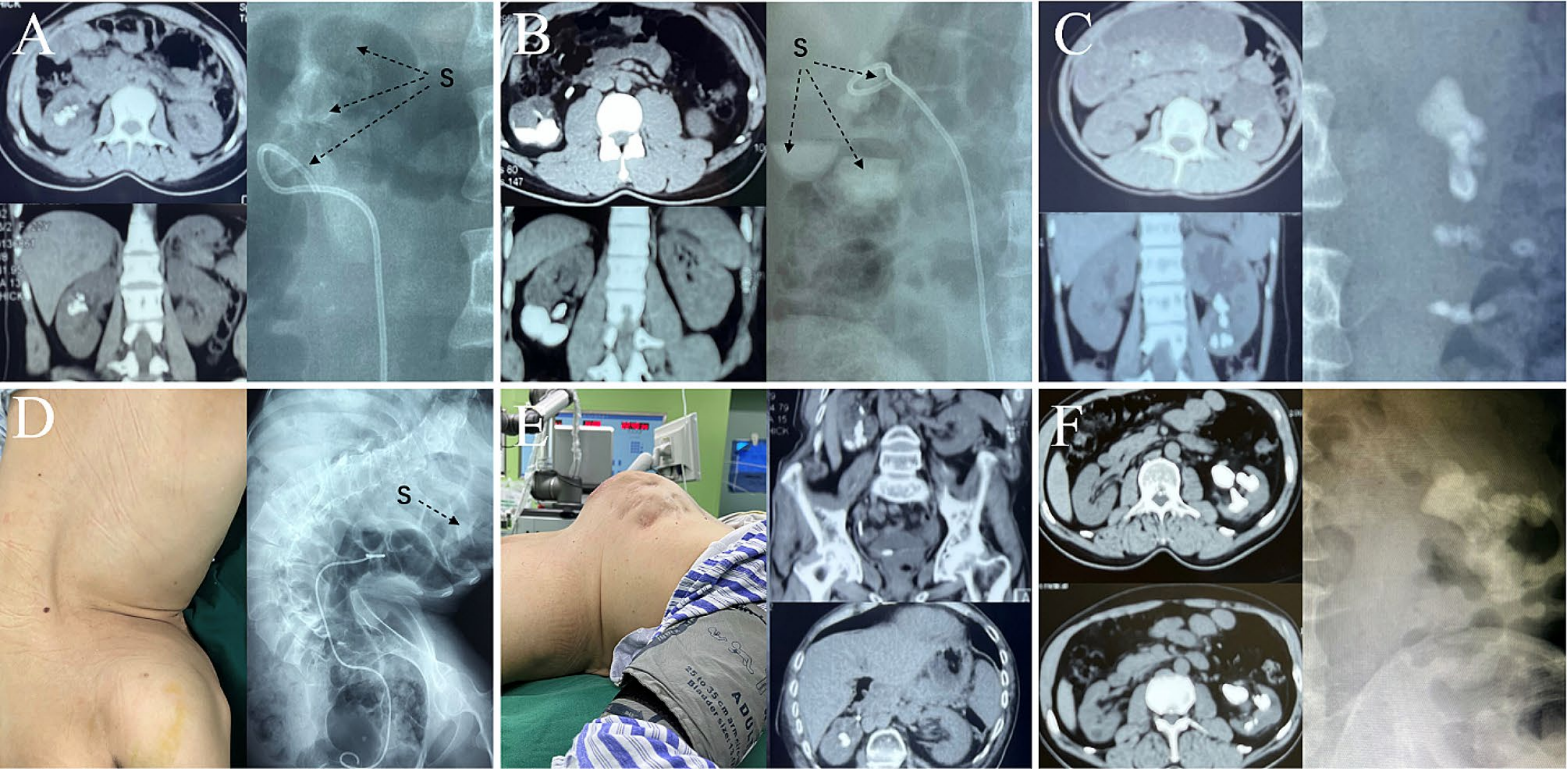
The preoperative abdominal non-contrasted computed tomographic (NCCT) and plain kidney-ureter-bladder (KUB) films of six enrolled cases with complex kidney stones (CKS). (**A**) Case 3, a partial staghorn stone with multiple stones in medium calyceal diverticulum, was assessed as Guy’s Stone Scores (GSS) grade III; (**B**) Case 11, multiple stones in the renal pelvis and lower calyceal diverticulum, was assessed as GSS grade III; (**C**) Case 8, a partial staghorn stone with multiple stones in lower calyceal diverticulum, was assessed as GSS grade III; (**D**) Cases 6, a stone in the pelvis of a solitary ectopic thoracic kidney (ETK) with a severe congenital scoliosis deformity, was assessed as GSS grade IV; (**E**) Case 9, multiple stones in the pelvis and lower calyceal diverticulum of an ETK with a severe rachiterata caused by spinal tuberculosis, was assessed as GSS grade IV; (**F**) Case 4, a complete staghorn stone, was assessed as GSS grade IV. Unclear high-density shadows of stones are denoted by black arrows in plain KUB films. S = stone

To prove technological superiorities, a consecutive series of 15 CKS cases (GSS grade III-IV) was enrolled in Xinjiang Kezhou People’s Hospital from October to November 2021. The GSS were assessed based on preoperative abdominal non-contrasted computed tomographic (NCCT) and plain kidney-ureter-bladder (KUB) films [6, 14]. GSS grade III denotes multiple stones in a patient with abnormal anatomy, or stones in a calyceal diverticulum, or a partial staghorn stone (Fig. 1A-C); GSS grade IV denotes a complete staghorn stone, or any stone in a patient with spina bifida or spinal injury [6] (Fig. 1D-F). No case had coagulation disorders or unacceptable anesthetic/operative risks assessed by American Society of Anesthesiologists (ASA) physical status score [15].

### Preoperative management

A mid-stream urine culture was performed before surgery [1, 7, 8]. For cases with a positive culture, a 5-day antibiotic was administered according to antibiogram findings; for cases with a negative culture, a prophylaxis antibiotic according to local prevalent antibiogram was administered 30 min prior to operation [2, 7, 8]. In patients with obvious obstruction, a double-J stent drainage was performed for several days [1, 2, 7].

### Surgical equipment and procedures

#### Surgical equipment

**Fig. 2:**
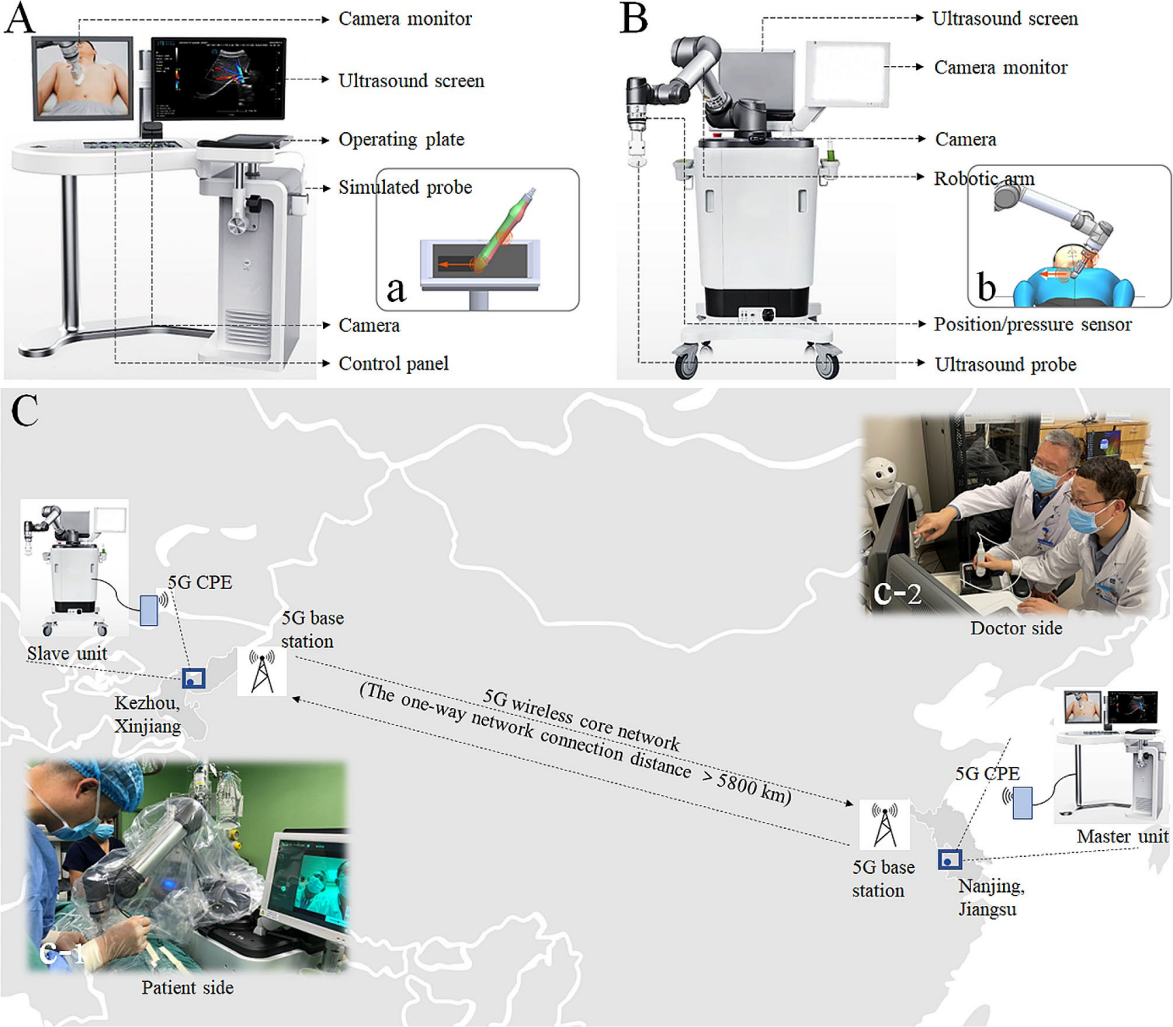
The tele-assistance platform for percutaneous nephrolithotomy (PCNL) mainly consists of the robot-assisted teleultrasound diagnostic system (RTDS) and 5G wireless core network. (**A**) the master unit of RTDS in doctor side; (a) the simulated probe and operating plate with position and pressure sensors in doctor side; (**B**) the slave unit of RTDS in patient side; (b) the ultrasound probe and robotic arm with position and pressure sensors in patient side; (**C**) the configuration of a tele-assistance platform powered by 5G network; (c-1) an intraoperative photo of PCNL guided by 5G-powered RTDS in Kezhou, Xinjiang; (c-2) a photo of online experts providing intraoperative tele-assistance in Nanjing, Jiangsu. CPE = customer premise equipment

The RTDS (MGIUS-R3, Huada Cloud-shadow Medical Technology Co., Ltd., China), used for tele-assistance, consists of a doctor-side unit (master unit, Fig. 2A) and a patient-side unit (slave unit, Fig. 2B). The slave unit is placed in the operating room of Xinjiang Kezhou People’s Hospital (Fig. 2c-1) and the master unit is in Internet Diagnosis and Treatment Center of First Affiliated Hospital of Nanjing Medical University (Fig. 2c-2). A master-slave control subsystem of RTDS is used to guarantee real-time consistency of scan-motions and contact-force between the doctor-side simulated probe and the patient-side robot-probe via several sensors (Fig. 2a, b) [10, 16]. The interactive audio-visual subsystem of RTDS allows intraoperative monitoring and communication between two sides [10, 17]. The 8/9.5 F ureteroscope (Karl Storz, Germany) and the holmium laser generator (Accu-Tech Co. Ltd., Beijing, China) with diameter 600µM optical fiber are used for stone removal.

#### Network connection and data transmission

The connection between the doctor and patient sides of RTDS is realized via a public 5G wireless core network (China Mobile Communication Corp., China) (Fig. 2C). Customer premise equipment (CPE) act as the terminal receivers of wireless signal generated by 5G base stations. Real-time dynamic images of teleultrasound first are compiled into corresponding byte codes at patient side, and then transmitted from CPE to the nearest base station via an encrypted data stream. Through 5G network, encrypted data is transmitted to the doctor-side base station and ultimately decoded to obtain patient’s ultrasound images at doctor side [10, 16, 17] (Fig. 2C). In turn, the scan-motion data of online experts is sent back by the same above mechanism to drive the robot-arm of RTDS at patient side.

#### Tele-assistance and surgical procedures

**Fig. 3:**
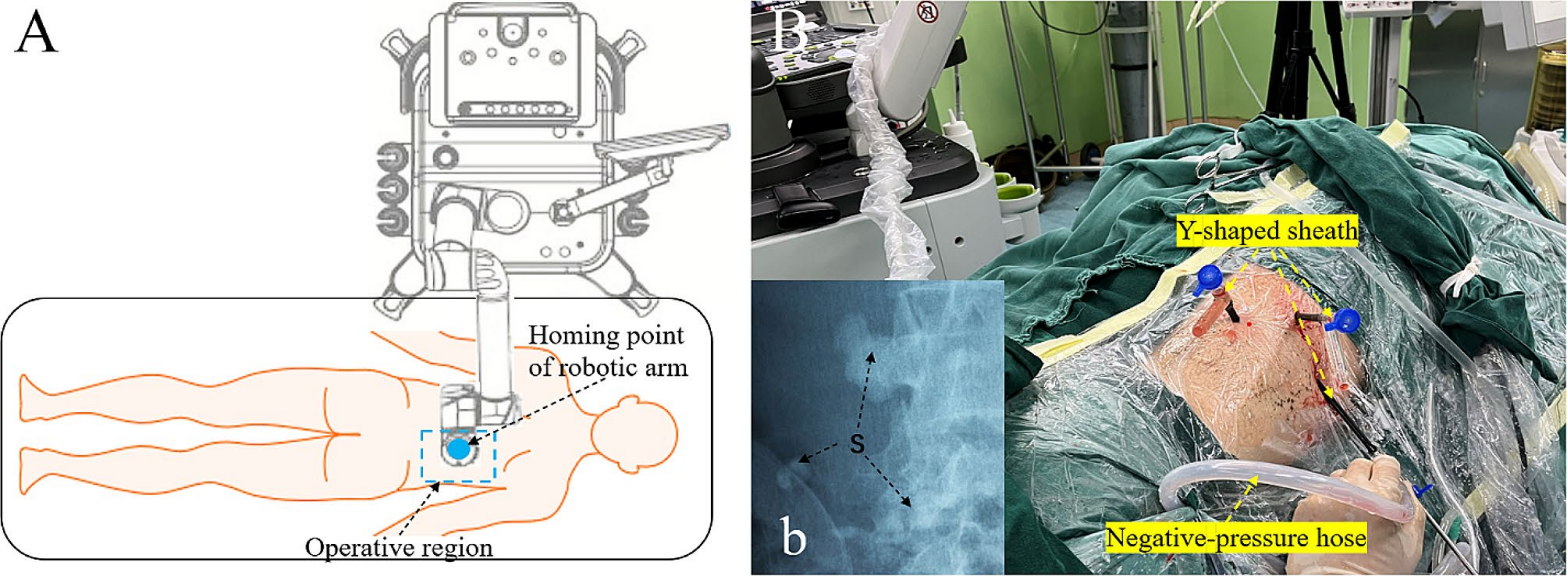
The intraoperative placement diagram of a slave unit in patient side and the establishment of three percutaneous renal accesses with continuous negative-pressure suction. (**A**) a slave unit of robot-assisted teleultrasound diagnostic system (RTDS) is placed on the opposite side of operative region and the homing point of robotic arm is located directly above the pre-formulated puncture site; (**B**) an intraoperative photo of three accesses established for an enrolled complex kidney-stone (CKS) patient, Case 5; (b) the preoperative plain kidney-ureter-bladder (KUB) film showed a partial staghorn stone with multiple stones in lower calices, assessed as Guy’s Stone Scores (GSS) grade III. Unclear high-density shadows of stones are denoted by black arrows in the plain KUB film. S = stone

Under general anesthesia, a patient was first placed in a lithotomy position for preplacing a 6 F double-J stent in the operation-side ureter, and then changed to a prone position for the tele-assistance PCNL. The slave unit of RTDS was placed on the opposite side of operative region and the homing point of robot-arm was located directly above the pre-formulated puncture site [10, 16, 17] (Fig. 3A, Supplemental Video. 1). After the slave unit was successfully connected with the master unit, a network test was carried out to ensure that the total delay was < 200ms and packet loss rate was < 5% [9, 11, 12].

After 500 ml saline was perfused into the bladder via a pre-indwelled Foley catheter, experts in Nanjing manipulated a simulated probe on the special operating plate of the master unit to drive the patient-side robot-arm from a straight-line distance of 3,900 km away. Real-time scan-motions and force magnitude were replicated by a 3.5-MHZ ultrasound probe of the robot-arm in patient side (Fig. 2a, b). According to dynamic teleultrasound images transmitted by 5G network, online experts precisely chose the best puncture point and step by step instructed local operators to continuously adjust the position, direction and depth of an 18-gauge needle-tip by the interactive audio-visual subsystem [12]. After successfully puncturing into the target calix through its fornix, experts continued to monitor the retention of an 0.035-inch J-tipped guidewire as well as each dilation-depth of expanders in order from 8 to 16 F [7, 8, 12]. Finally, a 14 or 16 F Y-shaped sheath with a negative-pressure hose was reserved to establish a vacuum-assisted access [12, 18]. (Figure 3B and b, Supplemental Video. 1)

After stones in all visual fields of an established access were fragmented and cleaned up by Holmium-laser lithotripsy with continuous negative-pressure suction, the probability of residual stones was determined by experts via teleultrasound examination. If residual stones were found, the second and even the third access would be established via the above procedure; if not, a 12 or 14 F nephrostomy tube was indwelled [7, 8, 12]. (Fig. 3B, Supplemental Video. 1)

### Perioperative evaluations

The length, width and height of stones were measured on NCCT images with digital calipers. Total stone volume (TSV) of a staghorn stone or multiple stones as well as residual stone volume (RSV) was calculated as the sum of each partial volume using the following formula: length× width× height × π × 1/6 [19]. The network-connecting parameters during each tele-assistance were monitored and recorded by an appointed engineer. Subjective evaluations for each communication and operation were investigated from both online experts and local operators via two 5-point scales (Table 2). A successful tele-assistance was defined as successfully guiding to establish all preplanned accesses for a case. Puncture-access ratio was defined as the guided puncture times for successfully establishing an access.

Complications were recorded according to Clavien-Dindo grading system [20]. SF status was defined as no radiological evidence or only the presence of ≤ 4 mm asymptomatic fragments [14, 21], and residual fragments of ≤ 4 mm were not counted into RSV. Stone clearance ratio (SCR) was calculated via the formula: (TSV- RSV)/TSV× 100%. According to RSV, the need for additional procedures (such as extracorporeal shock-wave lithotripsy, secondary PCNL or flexible ureteroscopy lithotripsy [fURL]) was determined by the principal investigator (Dr. Jie Yang). Data was analyzed using SPSS software v.19 (IBM, NC, USA).

### Follow-ups

All cases accepted a uniform follow-up including a review of inpatient and outpatient records and a NCCT examination for evaluating one-session SF rate and SCR on the postoperative 2nd day. The NCCT scanning was repeated at the 1st, 3rd, 6th, 9th or 12th month after the first operation of PCNL until residual fragments of > 4 mm were not detected. When residual fragments of > 4 mm were not detected, the NCCT scanning of next time point would not be conducted. If residual fragments of > 4 mm were found, we would carry out additional procedures for management of residual fragments. The clinical follow-up endpoint was determined in view of the evaluation of SF status and double-J stent removal; the research follow-up endpoint was scheduled to be at least one year after surgery.

## Results

### Demographics and clinical characteristics

In the current cohort, there were four patients (26.7%) suffering from severe obesity (body mass index [BMI] > 30 kg/m^2^) and two (13.3%) in anesthetic high-risk (ASA III-IV). A review on patient’s medical records showed that eight patients (53.3%) underwent a previous failure of kidney-stone surgery including four PCNL cases, three fURL and one pyelolithotomy. Preoperative NCCT and plain KUB films revealed that five patients (33.3%) were assessed as GSS grade IV, five (33.3%) had a large stone burden (TSV > 6 cm^3^) and six (40.0%) had an extremely high-density stone (CT value > 1200HU). Four patients (26.7%) had a notable renal-related anatomical abnormalities including one horseshoe kidney case, one severe nephroptosis, one ectopic thoracic kidney (ETK) with a severe rachiterata caused by spinal tuberculosis and one solitary ETK with a severe congenital scoliosis deformity. (Table 1)

**Table 1:** Demographics and clinical characteristics

Variables	Results
Total cases, *n*	15
Age, *yr, median (IQR)*	42 (33)
Gender, *n (%)*	
Male	6 (40)
Female	9 (60)
Body mass index, *kg/m*^*2*^	23.9 (9.7)
Anesthesiologists Society of America score, *n (%)*	
I	8 (53.3)
II	5 (33.3)
III	1 (6.7)
IV	1 (6.7)
Preoperative renal function, *n (%)*	
Normal	14 (93.3)
Abnormal	1 (6.7)
Guy’s Stone Scores grade, *n (%)*	
III	10 (66.7)
IV	5 (33.3)
Total stone volume, *cm*^*3*^, *median (IQR)*	4.7 (2.8)
Stone density, *HU, median (IQR)*	1044 (490.6)
Previous extracorporeal shock wave lithotripsy, *n (%)*	
No	8 (53.3)
Yes	7 (46.7)
Previous kidney-stone surgery^a^, *n (%)*	
No	7 (46.7)
Yes	8 (53.3)
Presence of hydronephrosis, *n (%)*	
No	6 (40)
Yes	9 (60)
Mid-stream urine culture, *n (%)*	
Negative	8 (53.3)
Positive	7 (46.7)
Renal-related anatomical abnormality^b^, *n (%)*	
No	11 (73.3)
Yes	4 (26.7)

^a^ Including flexible ureteroscopy lithotripsy, percutaneous nephrolithotomy or pyelolithotomy;

^b^ Including solitary kidney, horseshoe kidney, notable nephroptosis, ectopic thoracic kidney caused by scoliosis deformity or rachiterata;

HU = hounsfield unit; IQR = interquartile range

### Network-connecting quality and subjective evaluations

The one-way network connection distance was > 5,800 km. Median data-transmission rate reached 748 Mb/s (interquartile range [IQR] = 107) with median packet loss rate of 1.4% (IQR = 0.4). Median total delay was 177 ms (IQR = 2), primarily due to a mechanical-response delay of the slave unit, and median round-trip delay was only 13 ms (IQR = 2). (Table 2)

**Table 2:** Intraoperative network-connecting parameters and subjective evaluation investigated both from online experts and local urologists

Objective or subjective parameters	Results
Latency time, *ms, median (IQR)*	
Round-trip delay (t1)	13 (2)
Robot servo-period delay (t2)	6 (0)
Robot mechanical-response delay (t3)	121 (0)
Ultrasound-imaging delay (t4)	19 (0)
Video codec delay (t5)	18 (0)
Total delay (T)^a^	177 (2)
Data-transmission rate, *Mb/s, median (IQR)*	748 (107)
Packet loss rate, *%, median (IQR)*	1.4 (0.4)
Online experts’ evaluations, *n (%)*	
Satisfaction for doctor-side ultrasound image quality (4–5)^b^	14 (93.3)
Satisfaction for driving patient-side robot-arm (4–5)^b^ Satisfaction for total delay (4–5)^b^	15 (100) 15 (100)
Satisfaction for audio-visual communication (4–5)^b^	15 (100)
Confidence for completing tele-assistance (4–5)^c^	15 (100)
Local urologists’ evaluations, *n (%)*	
Satisfaction for robot-arm probe guidance (4–5)^b^	14 (93.3)
Satisfaction for audio-visual communication (4–5)^b^	15 (100)
Satisfaction for total delay (4–5)^b^	15 (100)
Confidence for establishing accesses (4–5)^c^	12 (80)
Confidence for completing stone removal (4–5)^c^	11 (73.3)

^a^ T = t1 + t2 + t3 + t4 + t5;

^b^ 1, very dissatisfied; 2, dissatisfied; 3, moderate; 4, satisfied; 5, very satisfied;

^c^ 1, very low; 2, low; 3, moderate; 4, high; 5, very high;

IQR = interquartile range

Subjective evaluations in Table 2 revealed high satisfaction with doctor-side ultrasound images as well as patient-side probe guidance, except a 3-point (moderate) rank was given from both sides for an obese female with a BMI of 36.7 kg/m^2^. No perceptible delay for driving robot-arm or audio-visual communication was fed back. The confidence of online experts was self-assessed at a high or very high rank in all cases. In contrast, the confidence for local operators to establish accesses was 80%, and a 3-point rank was rated in two ETK patients and one obese patient. The confidence for stone removal was only 73.3%, and local operators gave a 3-point rank in four patients because of large stone volumes or scattered distribution.

### Operative outcomes

Total 28 accesses were established via 36-time guided punctures, and repeated punctures were performed in six accesses. All cases underwent a successful tele-assistance for establishing adequate accesses to complete stone removal. No complications of grade III-V were found and two patients (13.3%) underwent a complication of grade II because of postoperative hemostatic usage, one of them (6.7%) requiring transfusion. One case (6.7%) required readmission because of persistent hematuria on the 3rd day after discharge. Four patients (26.7%) suffering from staghorn stones did not obtain one-session SF status despite three of them with three accesses established and the other one with two. But the calculation on RSV revealed a high one-session SCR (range: 92.5–98.3%), three of the four cases obtained SF status at postoperative 3-month end and the other one at 6-month end via one to three additional procedures. (Table 3)

**Table 3:** Operative outcomes and follow-up

Variables	Results
Cases with a successful tele-assistance, *n (%)*	15 (100)
Number of established accesses, *n (%)*	
1	6 (40)
2 3	5 (33.3) 4 (26.7)
Puncture-access ratio, *median (IQR)*	1 (0.5)
First-puncture access-success rate, *n (%)*	22 (78.6)
Operative time, *min, median (IQR)*	60 (45)
Hemoglobin drop, *g/dl, median (IQR)*	0.6 (0.7)
Complications, *Clavien-Dindo grading* [21], *n (%)*	
Grade I	5 (33.3)
Grade II	2 (13.3)
Additional procedures, *n (%)*	
ESWL	4 (26.7)
Second PCNL FURL	1 (6.7) 2 (13.3)
Postoperative hospitalization, *d, median (IQR)*	4 (1)
Readmission, *n (%)*	1 (6.7)
Clinical follow-up time, *mo, median (IQR)*	1 (2)
Research follow-up time, *mo, median (IQR)*	13 (1)
Postoperative SF rate, *n (%)*	
One-session SF rate	11 (73.3)
1-month SF rate	11 (73.3)
3-month SF rate	14 (93.3)
6-month SF rate	15 (100)

ESWL = extracorporeal shock-wave lithotripsy; FURL = flexible ureteroscopy lithotripsy; IQR = interquartile range; PCNL = percutaneous nephrolithotomy; SF = stone-free.

## Discussion

In remote regions, to meet the demand on high-quality medical services has always been a world-class problem because the improvements of staff experiences and skills as well as infrastructures cannot accomplish in one move [9]. Recently, network consultation, online prescribing and even imaging tele-examinations are becoming popular [10, 16, 17], which partially solves this problem. However, telesurgery, another subfield of telemedicine, is still just starting out for the requirements of higher network-connecting quality and advanced robot-operating system [9]. Since 1995, several attempts have been made worldwide to perform telesurgeries in various fields, such as neurosurgery, orthopedic surgery and endoscopic surgery. In 1995, Rovetta et al. conducted the pioneering remote transrectal ultrasound-guided prostate biopsy [22]. The first true intercontinental telesurgery took place in 2001, when surgeons in New York successfully performed a laparoscopic cholecystectomy using the ZEUS surgical robot on experimental pigs located in Strasbourg by utilizing a specially laid submarine cable for data transmission, and the straight-line distance was approximately 7,000 km. The procedure lasted for 45 min with an overall operant and feedback delay of 155 ms [23]. In 2005, Sterbis et al. utilized a commercial wired network to conducted a remote laparoscopic right nephrectomy. The maximum linear distance between the surgeon and the experimental pig was about 3,800 km, while the operation and feedback delay reached up to 900 ms [24].

5G technology is one of main requirements for the digital future [9]. In theory, it can offer peak transmission rate of 10-20Gb/s and latency time of < 1ms while improving data security and privacy [9]. Benefiting from 5G advantages, our median round-trip delay was only 13ms despite the one-way network-connecting distance between Kezhou and Nanjing > 5,800 km. Median total delay of our platform was 177ms, mainly composed of a mechanical-response delay of 121ms, and no perceptible delay was fed back. Subjective evaluations from online experts and local operators both showed high acceptability for the technology. Consistent with other reports [9, 11], our study also proves that a total delay of < 200ms can meet the demand of such an intraoperative tele-assistance.

Despite a high proportion of surgical difficulty-related factors including severe obesity (26.7%), anesthetic high-risk (13.3%), previous failure of kidney-stone surgery (53.3%), GSS grade IV (33.3%), large stone-burden (33.3%), no hydronephrosis (40%) and notable renal-related anatomical abnormalities (26.7%), a successful tele-assistance was obtained in all cases. The first-puncture access-success rate was 78.6% with a one-session SF rate of 71.3% and without complications of grade III-V. These encouraging results should be attributed to intraoperative assistance provided by online experts, which helped to directly solve multiple difficulties easily encountered by a beginner.

In a classic access-establishing step via ultrasound guidance, the first difficulty is lack of experience on designing access sites and puncture angles [8, 21, 25]. It is usually the key to determine whether an access can remove as many stones as possible while not to swing the sheath too much [21]. Under the current technology, online experts can give detailed advices by the interactive audio-visual subsystem, thus the experience-based barrier, which hindering PCNL popularization, will be broken down. The second difficulty is incompetent skills of operators including scan-motions unskilled or without proper force-magnitude when manipulating a probe; the misidentification of anatomic structures in ultrasound images, especially in patients with abnormal anatomies or severe obesity; unable to puncture into the target calix just through its fornix or failing to identify whether the needle-tip entering the target calix, especially in patients without hydronephrosis [7, 8, 26–28]. In our approach, the 5G-powered master-slave control subsystem permits the scan-motions with contact-force of online experts to be real-time replicated to the patient-side probe. Meanwhile, dynamic teleultrasound images transmitted by 5G network allow experts to accurately judge, guide and monitor the whole process of access establishment. Under the constant advice and guidance of experts via the communication subsystem and robot-arm of RTDS, local operators can make delicate adjustments and achieve a correct needle-tip orientation with an optimal track even in the presence of anatomical deformities or severe obesity. Finally, the skill-based barrier of PCNL will also be completely removed.

In addition to no exposure to ionizing radiation, several advantages deserve to be mentioned in our current tele-assistance. The first is that the robot-arm of RTDS greatly facilitates puncture operating. In a classic ultrasound-guided procedure, it is hard to maintain a needle-tip track always within the scanning plane when an operator holds a probe in one hand to guide and clenches a needle in the other to puncture [21]. In our procedure, local operators can puncture with both own hands and the probe is not easy to move and skid under the robot-arm control, which ensures the stability of ultrasound guidance (Fig. 2c-1). The second advantage is that the robot-arm probe provides stable real-time monitoring in the dilation step instead of blind operating based on personal experience and feeling. During dilation, it is hard to accurately control the proper depth of each expander because large-size expanders tend to push the kidney down [21, 27–29]. In our procedure, the teleultrasound probe, fixed above the puncture site by a robot-arm, makes the course semi-visualized and greatly increases the safety and the success rate of dilation. The third strong point is the re-examination for residual stones executed by online experts before the end of PCNL, which can obviously improve one-session SCR. In the current cohort, one-session SF rate was 73.3% despite median TSV up to 4.7cm^3^ (IQR = 2.8). Although four staghorn-stone cases didn’t obtain a one-session SF status, the calculated one-session SCR impressively reached a high range from 92.5 to 98.3%. Of course, the satisfactory stone-removal efficacy should also be partly attributed to the application of Y-shaped sheath with continuously negative-pressure suction (vacuum-assisted PCNL), which is the recent evolution of PCNL armamentarium. Vacuum-assisted PCNL has been associated with higher SF rate than classic PCNL [18].

Compared to wired networks that necessitate specific network cables or optical cables, 5G networks enable enhanced device mobility and eliminate geographical limitations leaded by network optical cables. Remote assisted surgery can be conducted between two locations through a wireless connection utilizing only a dedicated 5G CPE as a signal relay station and amplifier. The improved devices mobility facilitated by 5G wireless networks allow for remote surgeries in areas where cable infrastructure is scarce, such as space, battlefields, disaster zones, and mountainous regions [30]. Meanwhile, some difficulties need to be overcome when using this technology. First, it is essential to conduct thorough testing of network speed, bandwidth, data transmission delay, and packet loss rate between the two locations prior to the operation. Where feasible, a wired network connection can be employed as a backup scheme to ensure uninterrupted and reliable connectivity during the operation [31]. Second, the doctor’s visual perception during the procedure solely relies on real-time two-dimensional images captured by a camera positioned at the patient’s side. Therefore, depth perception of doctor remains limited. There is a disparity between using a simulated probe on the doctor-side unit and performing an actual B-ultrasound examination. Therefore, doctors need more practice of the RTDS [32].

Currently, the prospective study is in IDEAL stage 1, mainly aiming at the proof of concept and feasibility, and has some limitations. Owing to a small sample-size of 15 cases, exploration on some important problems associated with this novel technology is far from sufficient such as complication rates, potential ethical or legal problems, secondary procedure choice and follow-up outcomes. The most challenging one is potential legal problems [9, 11]. For example, when severe complications occur, how to identify the medical accident liability between online experts and local operators is still unknown. In this scenario, relevant laws and regulations must be made or improved as soon as possible [9]. Moreover, our tele-assistance approach is also a superb “hand to hand” teaching mode for local urologists, so another limitation is that the technical improvement of local operators and their learning-curve shortening is not assessed after they have been tele-assisted for several times. Thus, the tele-assistance technology needs to be applied to more patients in multiple centers for further verification. These preliminary encouraging findings need to be validated in further investigations. We plan to demonstrated the satisfactory outcomes and reliable safety of this technology by different study designs based on IDEAL stages.

## Conclusions

The current technology based on the platform of 5G-powered RTDS can provide a high-quality tele-assistance with establishing proper accesses during PCNL. It will break down a personal competence-based barrier to endow PCNL with more popular utilization. Our study has shown satisfactory outcomes and reliable safety of this technology in a CKS cohort. We expect the innovatory technology to play an enlightening role in meeting the demand on high-quality medical services in regions with undeveloped public health.

### Electronic supplementary material

Below is the link to the electronic supplementary material.

Supplementary Material 1

## Data Availability

Data are freely available at a data archive on http://www.chictr.org.cn (ChiCTR2200065849).

## Declarations

### Statement for CONSORT guidelines

Our study adheres to CONSORT guidelines.

## Abbreviations

CKS: Complex kidney-stone
GSS: Guy’s Stone Scores
PCNL: Percutaneous nephrolithotomy
5G: 5th generation mobile communication technology
RTDS: Robot-assisted teleultrasound diagnostic system
KUB films: Plain kidney-ureter-bladder films
TSV: Total stone volume
RSV: Residual stone volume
SCR: Stone clearance ratio
fURL: Flexible ureteroscopy lithotripsy
BMI: Body mass index
IQR: Interquartile range
